# 89. Cytomegalovirus (CMV) Serostatus Reassessment for Accurate CMV Risk Stratification in CMV Seronegative Kidney Transplant Candidates

**DOI:** 10.1093/ofid/ofaf695.035

**Published:** 2026-01-11

**Authors:** Ajmeet K Pama-Ghuman, Puneet Sood, Monica Fung, Lakshin Kumar, Garrett R Roll, Anna Mello, Abhijit Limaye

**Affiliations:** University of California, San Francisco (UCSF), Kingsburg, CA; UCSF, San Francisco, California; University of California San Francisco, San Francisco, California; University of California San Francisco, San Francisco, California; UCSF, San Francisco, California; UCSF, San Francisco, California; Univ of California San Francisco, san francisco, California

## Abstract

**Background:**

Accurate recipient CMV serostatus at time of transplant is critical to guide post-transplant CMV preventive strategies. However, seroconversion can occur between the several-year wait time between listing (time of initial CMV serostatus testing) and transplant, potentially leading to misclassification unless serology is reassessed more proximate to transplant. The optimal timing for CMV serostatus reassessment is unknown. This quality improvement/assurance study assessed adherence to a programmatic recommendation to reassess CMV serostatus within 6 months of transplant.

**Figure 1.**
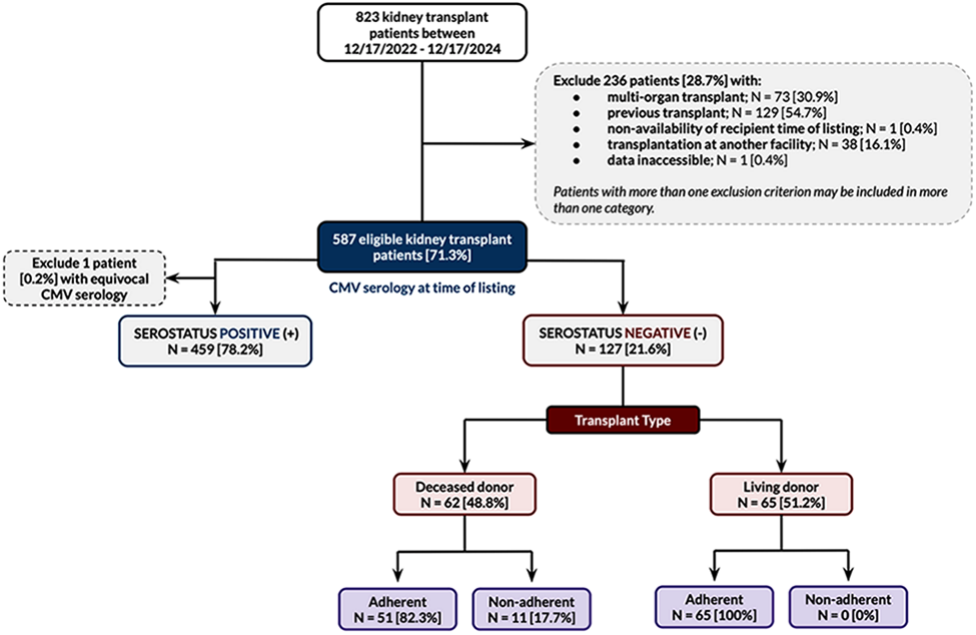
Summary of participants and results

**Table 1.**
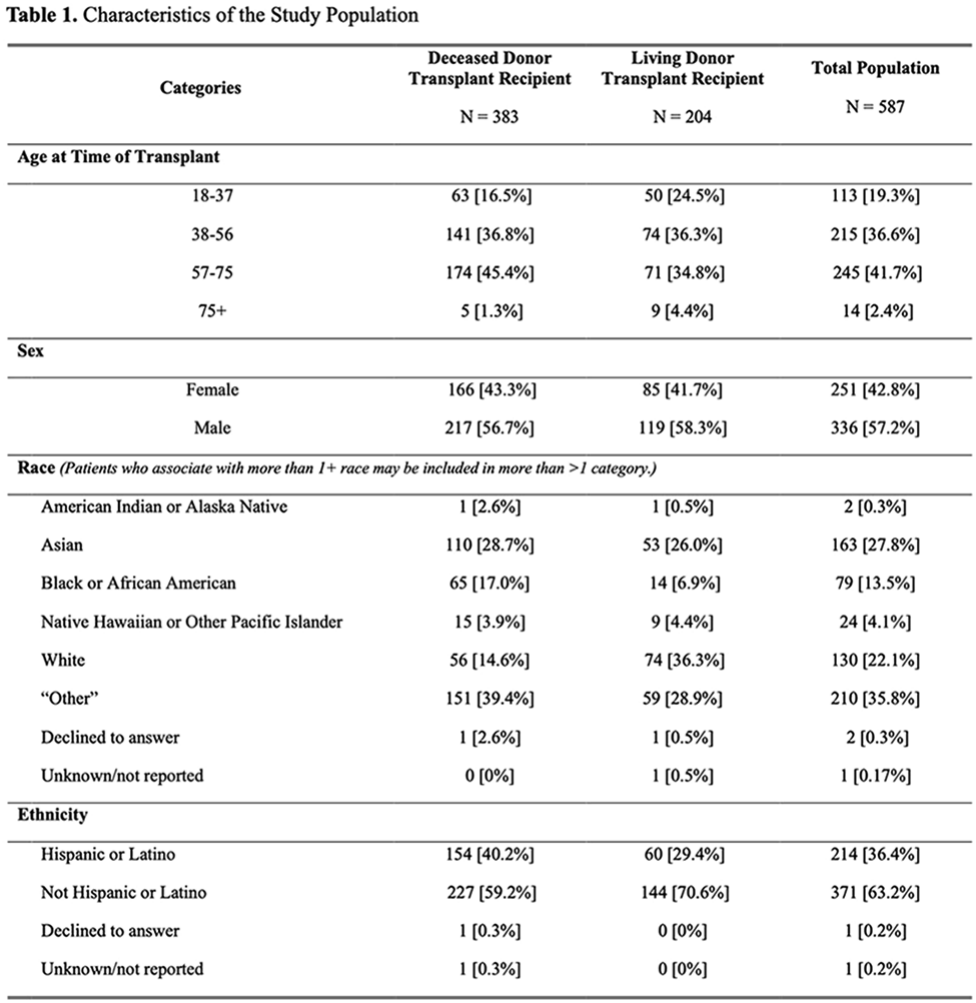
Characteristics of the Study Population Table 1 summarizes the various characteristics tracked in the study population, including transplant type, age, sex, race, and ethnicity.

**Methods:**

Adherence to CMV serology reassessment within 6 months of transplant among CMV seronegative patients at listing was retrospectively assessed at a single large US academic transplant center in adult recipients of a first transplant between 12/17/2022-12/17/2024. Baseline and transplant factors were compared between adherent and non-adherent groups and significance between proportions was assessed by Chi-square or Fisher’s exact test, with p-values < 0.05 considered significant. The incidence of seroconversion between the time of listing and transplant was assessed among those who had CMV serology reassessed.

**Results:**

Among 823 adult kidney transplant recipients, 587 (71.3%) met eligibility criteria and 127 (21.6%) were CMV seronegative. The median (interquartile range) time to transplant was 3.75 [1.45 – 6.08] years and was longer for deceased (5.23 [2.89 – 7.44] years) compared to living donor transplants (1.51 [0.76 – 2.74] years). Overall adherence was 91.3% (116/127) and was significantly higher among living (65/65 [100%]) vs deceased donor transplants (51/62 [82.3%]), p < 0.01). No demographic or transplant-related variables were significantly associated with adherence (p >0.05 for all). Seroconversion occurred in 2 of 124 patients (1.6%) at 8.4 and 1.7 years (incidence of 0.43 per 100 person-years of follow-up).

**Conclusion:**

Adherence to a programmatic recommendation for CMV serostatus assessment within 6 months of transplant was >80% overall and significantly higher among living vs deceased donor transplant recipients. The low incidence of CMV seroconversion in this population allows for flexibility in the implementation of CMV serostatus reassessment based on feasibility.

**Disclosures:**

All Authors: No reported disclosures

